# Preparation of Seaweed Nanopowder Particles Using Planetary Ball Milling and Their Effects on Some Secondary Metabolites in Date Palm (*Phoenix dactylifera* L.) Seedlings

**DOI:** 10.3390/life13010039

**Published:** 2022-12-23

**Authors:** Amal A. Mohamed, Manal Y. Sameeh, Hossam S. El-Beltagi

**Affiliations:** 1 Chemistry Department, Al-Leith University College, Umm Al-Qura University, Makkah P.O. Box 21955, Saudi Arabia; 2 Plant Biochemistry Department, National Research Centre, 33 El-Behooth St., Dokki, Giza P.O. Box 12622, Egypt; 3 Agricultural Biotechnology Department, College of Agriculture and Food Sciences, King Faisal University, Al-Ahsa P.O. Box 31982, Saudi Arabia; 4 Biochemistry Department, Faculty of Agriculture, Cairo University, Giza P.O. Box 12613, Egypt

**Keywords:** Ball mill, date palm, DPPH method, date palm, flavonoids, polyphenols, seaweed, environmental health and safety

## Abstract

Due to their distinctive physicochemical characteristics, nanoparticles have recently emerged as pioneering materials in agricultural research. In this work, nanopowders (NP) of seaweed (*Turbinaria triquetra*) were prepared using the planetary ball milling procedure. The prepared nanopowders from marine seaweed were characterized by particle size, zeta potential, UV-vis spectroscopy, Fourier transform infrared (FT-IR) spectroscopy, transmission electron microscopy (TEM), scanning electron microscopy (SEM) and X-ray diffraction (XRD). When the seaweed nanopowder of *Turbinaria triquetra* was subjected to FT-IR analysis, it revealed the presence of different functional groups, including alkane, carboxylic acids, alcohol, alkenes and aromatics. Moreover, the methanol extract was used to identify the polyphenolic components in seaweed (NP) using high performance liquid chromatography (HPLC) and the extract revealed the presence of a number of important compounds such as daidzein and quercetin. Moreover, the pot experiment was carried out in order to evaluate the effects of prepared seaweed (NP) as an enhancer for the growth of date palm (*Phoenix dactylifera* L.). The date palm seedlings received four NP doses, bi-distilled water was applied as the control and doses of 25, 50 or 100 mg L^−1^ of seaweed liquid NP were used (referred to as T1, T2, T3 and T4, respectively). Foliar application of liquid NP was applied two times per week within a period of 30 days. Leaf area, number of branches, dry weight, chlorophylls, total soluble sugars and some other secondary metabolites were determined. Our results indicated that the foliar application of liquid NP at T3 enhanced the growth parameters of the date palm seedlings. Additionally, liquid NP at T3 and T4 significantly increased the photosynthetic pigments. The total phenolic, flavonoid and antioxidant activities were stimulated by NP foliar application. Moreover, the data showed that the T3 and T4 doses enhanced the activity of the antioxidant enzymes (CAT, POX or PPO) compared to other treatments. Therefore, the preparation of seaweed NP using the planetary ball milling method could produce an eco-friendly and cost- effective material for sustainable agriculture and could be an interesting way to create a nanofertilizer that mitigates plant growth.

## 1. Introduction

The widespread use of chemical fertilizers has negative impacts on soil deterioration, groundwater pollution and food contamination. As a result, during the last few years, several attempts have been made to replace chemical fertilizers with eco-friendly biofertilizers. Seaweeds are marine macroalgae which play a significant role in marine biological resources. They contain major and minor mineral elements, fatty acids, vitamins, cytokinins, auxins and abscisic acid [1,2]. In more detail, Irkin and Yayintas [1] stated that seaweeds are valuable products both nutritionally and medicinally. Their high mineral content and the beneficial sulfated polysaccharides have important value for human health. The primary class of bioactive compounds to reduce numerous chronic diseases including cancer, diabetes and cardiovascular diseases are seaweed polysaccharides. All of the known vitamins, chlorophylls, lignans and antioxidant compounds that may be beneficial to human health are rich in seaweeds. Seaweeds should receive a lot of attention in order to balance out the food function. Moreover, due to their significant nutritional value, much more attention should be given to different industry sectors produced from seaweed. Moreover, El-Beltagi et al., [2] reported that macroalgae extracts are currently employed as foliar sprays or are used in presoaking to promote plant growth due to the availability of numerous trace elements, vitamins, growth regulators or amino acids. Seaweed extracts have been widely used in agriculture to boost crop productivity. Moreover, they contain a highly valuable nutritional amount of several pharmaceutical compounds such as carrageenan, sulfated polysaccharides, polyphenols, alginate and amino acids [2]. Based on the range of bioactive compounds mentioned above in seaweeds, seaweeds have recently been heavily utilized in several biotechnological applications; they play a significant role in agriculture acting as biofertilizers and soil conditioners. When alginate oligosaccharide is extracted from the brown seaweed species and prepared in nanoparticle form, it exhibits significant biological activity [3]. In order to fully exploit the diversity and complexity of seaweed, one must be aware of the effects of the environment as well as have a thorough understanding of the biological variability. The content or diversity of seaweed metabolites are influenced by nutrient enrichment, reproductive status, phylogenetic diversity and time of collection [4]. Application of liquid seaweed extracts has been shown to increase seed germination in a different plant species, for example in Faba bean [5] and Lettuce [6]. The application of seaweed extract as a bio-stimulant has resulted in promising increases in nutrient uptake and tolerance to different environmental stresses in *Zea mays* plants [7].

The last few years have seen a huge advantage in nanotechnology as matter can now be modified at the macromolecular level to create nanometer-scale structures with unique features due to their tiny size with a diameter of <100 nm. Since the plant cell wall reduces the entry of nutrients into cells, nanoparticles with diameters smaller than the pores in the cell wall can readily pass through the pores. Through the leaves’ stomata or roots the influence of nanostructures resulted in the simpler absorption and improved nutrient penetration of the Jerusalem artichoke plant [8]. Nowadays, nanofertilizers (NFs) are considered useful in agriculture to improve growth and quality parameters because they supply nutrients to plants in a more available form which increases nutrient uptake and increases plant growth [9,10]. Accordingly, direct exposure of wheat plants to nano-chitosan NPK fertilizer induced an increased yield production [11]. Moreover, the advantages of nanofertilizers in terms of fruit quality, productivity, shelf life and lowering nutrient loss in soil after the period of crop harvest have been affirmed [12,13].

One of the most recent technologies that has attracted interest across several fields is nanoparticles (NPs). It is thought that the biological synthesis of NPs (using, for example, microorganisms such as bacteria, fungi and yeast, microalgae or plant extracts including macroalgae) is a secure, easy and environmentally friendly method that uses straightforward reducing agents rather than dangerous chemicals [14]. Numerous uses for nanoparticles (NPs) exist for preserving sustainable agriculture and healthy ecosystems as well as for resolving environmental and agricultural issues. This technology can increase crop yields, improve nutrient absorption from soils, decrease the use of pesticides and fertilizers and improve food quality and safety. There are two methods for creating NPs: bottom-up and top-down. In the first method, atoms, ions and molecules interact chemically, whereas in the second method, material fragments are broken down mechanically to produce the necessary nanoparticle sizes. The top-down method of NP manufacturing, which frequently leads to flawed surface structures, is not preferred since it produces particles with a different chemical content and more problems [15]. In this regard, other authors [16] used shrimp shells and squid pin waste as byproducts to prepare an eco-friendly nanofertilizer named chitosan (CS) and they evaluated the stimulation effects of the prepared nanofertilizer of chitosan on the growth of one plant named *Capsicum annum* L. cv. Cordoba. However, the *Capsicum annum* L. cv. Cordoba plant was subjected to foliar-applied chitosan with different concentrations of nanofertilizer (10%, 25%, 50% and 100%), and the results were compared to commercial chemical fertilizer and untreated control plants. The acquired results showed that, in comparison to the control and chemical fertilizer-treated plants, the nanocomposite with a 25% concentration considerably increased the growth, yield and harvest of *C. annuum*. This study showed that using a low-concentration eco-friendly preparation of waste composites as a foliar fertilizer could improve the growth and productivity of capsicum.

Nanofertilizers (NFs) are a cost-effective substitute for common chemical fertilizers that can sustainably improve world food production [17]. Ball milling is an efficient and environmentally friendly technology that modifies the molecular arrangement of the surfaces of some materials, as well as their crystal structures and electron arrangements. This leads to a variety of unique effects, such as the production of nanoparticles, which are not present in the original material. Ball-milled products have shown significant changes in physical and chemical properties such as solubility, bioavailability, distribution, absorption and extraction efficiency in the case of chick pea powder [18].

Date palm (*Phoenix dactylifera* L.) is one of the most significant economic crops that belongs to the family Arecaceae which is still the most widely grown and low expensive fruit in tropical regions, has a high nutritional value and is highly rich in antioxidants. The chemical composition of dates is variable due to various environmental factors such as growing region, climate, soil conditions, amount of fertilizer used during the growth stages and also the types of cultural practices [19,20]. The use of soil-applied nanofertilizer promoted date palm growth and production in comparison to the usage of conventional fertilizer [21]. In folk medicine, various portions of the date palm are used as a treatment for diseases such as fever, inflammation, nerve damage and loss of consciousness [22]. Moreover, the pharmacological characteristics of date palm fruits, including their anti-inflammatory, antioxidant and antimicrobial effects have been studied [23]. Recently, the possible use of date palm extract against COVID-19 was stated as well [24].

The physiological and biochemical mechanisms that occur from the application of a prepared nanofertilizer on the growth of the date palm have not been fully studied; therefore, this paper aimed to examine the effect of the foliar application of seaweed nanopowders (NP) on some of the physiological or biochemical parameters of date palm (*Phoenix dactylifera* L.) seedlings. Optimization of the ball milling technique to prepare nanopowders (NP) of seaweed (*Turbinaria triquetra*) was investigated. The date palm seedlings were treated with different concentrations of seaweed nanopowders. In response to the foliar application of seaweed nanopowders, some biochemical parameters of the date palm seedling were evaluated as well. 

## 2. Materials and Methods

### 2.1. Chemicals

Folin–Ciocalteu reagent, 96% sulfuric acid, standard glucose, 2,2-diphenyl-1-picrylhydrazyl, (DPPH), Butylated hydroxyltoluene (BHT) or aluminum chloride were purchased from Merck Company (Darmstadt, Germany). The other analytical standards of the monitored bioactive compounds by HPLC were supplied by Sigma-Aldrich (Milan, Italy). All other reagents used in this work were of the highest analytical grade purity or were purchased from Sigma chemicals, St. Louis, MO, USA.

### 2.2. Collection of the Seaweed Samples

**Figure 1:**
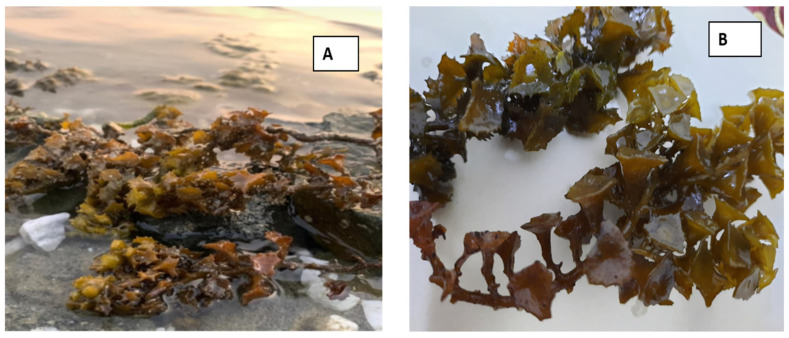
Seaweed image of *Turbinaria triquetra*: (**A**) view in the natural habitat at the beach of Al-Leith city, (**B**) view in the Lab.

Seaweed samples were collected from the Red Sea coast of Saudi Arabia at Al-Leith city, Makkah Province, during 2021 at depths ranging between 0.1 m and 0.3 m. They were collected in polyethylene bags; thereafter, they were transported to the lab. The seaweed was identified [25] under the light microscope according to its morphological characteristics with taxonomic references to *Turbinaria triquetra* and Figure 1 represents the image of *Turbinaria triquetra.*

### 2.3. Preparation of Nanopowders from Turbinaria triquetra Seaweed

**Figure 2:**
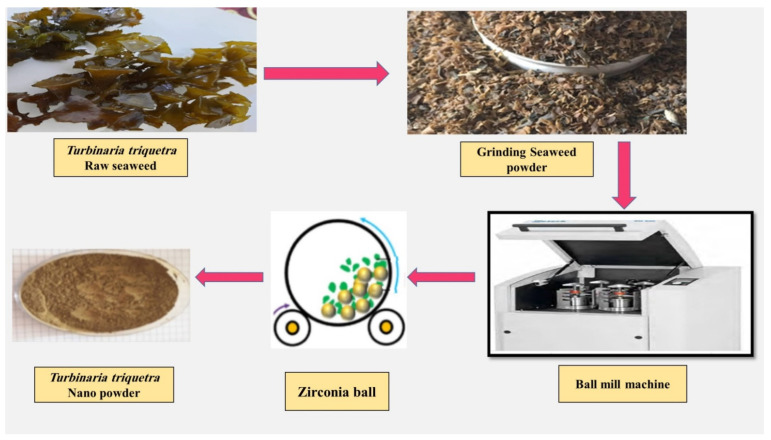
Schematic representation of the nanopowder ball milling process in the lab.

About 2000 g of seaweed material was washed multiple times with tap water to remove the dust and impurities or salts adhered to the outer surface, then finally it was washed with bi-distilled water. The cleaned seaweed was shade dried and powdered in a mixer grinder. The ground seaweeds were ball-milled with a planetary ball mill (Fritsch™ Pulverisette™ 6 Classic Line Planetary Mono Mill, Idar-Oberstein, Germany) with a steel jar of 250 mL volume. Ultrafine grinding was performed by mixing seaweed powder (using 100 g) and three zirconium balls of 10 mm along with two zirconium balls of 25 mm for 25 h, after that two extra zirconium balls of 25 mm were added and the grinding process was continued for an extra 10 h. The milling jar was rotated alternately in the forward or reverse direction at intervals of 2 min while the disk rotated at a speed of 350 rpm. Figure 2 represents the preparation steps of the seaweed nanopowders in the lab.

These steps were repeated until all the samples were milled by the ball mill. Ultimately, the prepared powder was kept in vials for further particle size assessment, zeta potential, TEM, SEM and XRD measurements as follows. 

### 2.4. Characterization of the Resulting Seaweed Nanopowders (NP)

#### 2.4.1. Particle Size or Zeta Potential 

Average diameter, size distribution or zeta potential of the samples were tested by utilizing a particle size analyzer, Malvern Zetasizer 3000 HSA (Nano-ZS, Malvern Instruments Ltd., Cambridge, UK). A sample of nanopowder was disseminated in bi-distilled water (as the solvent) dispersant at 25 °C. The sample was exposed to photon rays, which caused the particle to respond. The particle ray’s dispersion caused fluctuations in distribution intensity that were clumped in the targeted corner and were then detected by a sensitive detector. The sample suspension underwent sonication for about 60 min just before assessment [26]. 

#### 2.4.2. Fourier Transform Infrared (FT-IR) Spectra of the Seaweed Nanopowder of *Turbinaria triquetra*

Infrared spectra of the seaweed nanopowder of *Turbinaria triquetra* were obtained by a JASCO Fourier transform infrared spectrometer (FT-IR 6100, SN: A009061020) equipped with a TGS detector and a diffuse reflectance (DRIFT) accessory. The spectral data were processed with the IR solution Software Overview and Origin R 7SR1 Software, Spectra Manager version 2.

#### 2.4.3. UV Scan of the Nanopowder of *Turbinaria triquetra*

UV spectra of the water-diluted suspension of the nano-powder of *Turbinaria triquetra* were obtained using a spectrophotometer (JASCO V-730, Tokyo, Japan) and absorbance values of the peaks were scanned at 200–800 nm.

#### 2.4.4. TEM and SEM Characterization

Morphology and particle size of the nanopowder mediated by the *Turbinaria triquetra* extract were defined via TEM (HR-TEM, JEOL-JEM-2100) or SEM (Mira3 Tescan). The dilute suspension of nanopowders (100 mg/L) using bi-distilled water was sonicated for about 1 h prior to investigation to ensure the uniform dispersion of the particles. One or two drops of the sonicated suspension were dropped onto a 200-mesh size carbon-coated copper grid (Ted Pella, Redding, CA, USA) and left for drying in air before examination. Gold sputtering was performed using an Edwards S150 sputter coater. The atomic number sensitivity of the backscattered electron (BSE) was exploited to distinguish nanopowders with different chemical compositions. The determination was analyzed in the Nanotechnology and Advanced Material Central Lab, located in the National Research Centre Laboratory, Dokki, Giza, Egypt. 

#### 2.4.5. XRD Characterization

The XRD analysis of the sample was performed using a PAN analytical X-ray Diffraction equipment model X’Pert PRO with a Secondary Monochromator and Cu radiation (λ = 1.542 Å) at 45 K.V., 35 M.A. and a scanning speed of 0.04°/sec. The diffraction peaks between 2θ = 2° and 60°, corresponding spacing (d, Å) and relative intensities (I/I°) were obtained. The diffraction charts and relative intensities were obtained and compared with ICDD files.

### 2.5. HPLC Profile Determination

Seaweed extraction for HPLC analysis: A nanopowder of *Turbinaria triquetra* seaweed (1 g) was rinsed in 10 mL methanol 70% in glass bottles. Bottles were shaken for 24 h at room temperature in an orbital shaker (Heidolph—Unimax 2010-Germany). The extract was filtered through Whatman No. 1 filter paper; the residue was washed two times with an aliquot of methanol 70% and filtered through Whatman No. 1 filter paper. The net filtrate was adjusted to 10 mL with methanol 70%. HPLC analysis of the 70% methanol extract was carried out using HPLC (Agilent technologies, 1260 Infinity- Mundelein, IL, USA). A 4.6 mm × 250 mm i.d., 5 m, Eclipse C18 column was used for separation. Water (A) and 0.05% trifluoroacetic acid in acetonitrile (B) were the components of the mobile phase, which had a flow rate of 0.9 mL/min. The mobile phase was programmed consecutively in a linear gradient as follows: 0 min (82% A); 0–5 min (80% A); 5–8 min (60% A); 8–12 min (60% A); 12–15 min (82% A); 15–16 min (82% A); or 16–20 (82% A). The multi-wavelength detector was detected at 280 nm. Injection volume was 5 μL of the sample solutions and the column was kept at a constant temperature of 40 °C.

### 2.6. Elemental Composition of Seaweed Nanopowders

The element constituents of the seaweed nanopowders were characterized using Axios advanced, Sequential wavelength dispersive X-ray fluorescence spectrometry (WD-XRF) model (Malvern, UK), located in the National Research Centre Laboratory, Dokki, Giza, Egypt. 

### 2.7. Pot Experiment on the Date Palm Seedlings 

The pot experiment was conducted according to the previously described method [27] in order to evaluate the effect of the foliar application of the prepared seaweed nanopowders (NP) in liquid form on the growth of date palm (*Phoenix dactylifera* L. cv. Sukary) seedlings. The seedlings were a gift from a private farm at Al-Leith city, Makkah, KSA, during 2021. Uniform seedlings were selected for the experiment and were taken from healthy plants grown in the nursery at the 60-day growth stage. The nanopowders (NP) of seaweed were dissolved using bi-distilled water to prepare the different concentrations. The experiment comprised of four treatments of nanofertilizer solution (applying bi-distilled water as a control, 25, 50, 100 mg L^−1^ of seaweed liquid NP (which are referred to as T1, T2, T3 or T4, respectively). The experiment was carried out in triplicate and each treatment contained 5 pots (group), with each pot containing 1 seedling and 3 groups of repetitions. During the experiment, the liquid NP was applied two times per week within a period of 30 days. The nanopowders were foliar-applied on both sides of the leaves. After 30 days of the foliar application of nanopowder treatments, the samples were collected for the following analysis.

### 2.8. Determination of Morphological Parameters 

The following parameters were recorded: shoot height (cm), leaf area (cm^2^) and number of branches per plant. Moreover, fresh leaves were placed in a 70 °C oven for 48-h until a constant weight was reached, then the dry weight (g) of the leaves was measured [28].

### 2.9. Biochemical Parameters 

#### 2.9.1. Preliminary Phytochemical Screening

Two solvents with different polarities were used to extract the samples from various treatments, (ethanol and diethyl ether) for preliminary phytochemical screening such as alkaloids, terpenoids, steroids, tannins, saponins and flavonoids using the phytochemical tests described [29]. 

#### 2.9.2. The Content of Chlorophylls

Chlorophylls (Chl) were extracted by acetone (80% *v*/*v*) using a pestle and mortar, and then filtered. The chlorophyll content was measured in aliquot extracts using a spectrophotometer (UNICAM UV300—UK model (at 662, 644 and 470 nm and the absorbance readings were applied to equations published previously [30] in order to calculate the chlorophyll and total carotenoid contents. The contents of the pigments were calculated as mg g^−1^ fresh weight as follows:

Chl.*a* = 12.7 × O.D.662 − 2.69 × O.D.644 (1)

Chl.*b* = 22.9 × O.D.644 − 4.68 × O.D.662 (2)

Chl.*a+b* = 20.2 × O.D.644 + 8.02 × O.D.662 (3)

Carotenoids = (A470 − 1.28 (Chl.*a*) + 56.7 (Chl.*b*)/(256 × 0.906) (4)

#### 2.9.3. Determination of Total Soluble Sugar

The phenol sulfuric acid reagent method was utilized to measure the total amount of soluble sugars in the date palm-treated leaves [31]. Briefly, in a test tube, a 5% phenol solution and a 2 mL aliquot of a sample solution were combined. The liquid was then quickly added to 5 mL of pure sulfuric acid. The test tubes were vortexed for 30 s after standing for 10 min at room temperature, and then they were placed in a water bath at room temperature for 20 min to allow for color development. Then, at 490 nm, light absorption was measured using a spectrophotometer (UNICAM UV300—UK model). The preparation of reference solutions was the same as described above, with the exception that double distilled water was used instead of the 2 mL aliquot of the sample. The total soluble sugar was expressed as % dry weight.

#### 2.9.4. Preparation of Ethanolic Extracts for TPC, TFC and Antioxidant Activities

The collected leaf samples from each treatment were air-dried in the lab at room temperature and then ground to a powder. The pulverized samples (5 g) were macerated in 50 mL ethanol in an Erlenmeyer flask. Maceration was completed at an ambient temperature in plug vials with shaking for 24 h. on an orbital shaker (Heidolph—Unimax 2010- Berlin, Germany). The extracts were filtered through Whatman No. 1 filter paper. The pooled solution was evaporated under Rota-vapor (Heidolph- Berlin, Germany) at 40 °C. The resulting extracts were re-dissolved in methanol at a concentration of 1 mg/mL or utilized for further analysis. 

#### 2.9.5. Total Phenolic Content (TPC)

From each ethanolic extract, 200 μL was taken and completed to 3 mL with bi-distilled water then combined with 0.5 mL of Folin–Ciocalteu reagent. After mixing for 10 min, 2 mL of 10% (*w*/*v*) Na_2_CO_3_ was added or allowed to stand for another 30 min in the dark. The absorbance of the reaction mixture was determined at 650 nm with a spectrophotometer (UNICAM UV300—UK model). Total phenolic content was expressed as the mg/g GAE equivalent of the dry weight (d.w) of the extract [32]. 

#### 2.9.6. Total Flavonoid Content (TFC)

In brief, 0.5 mL of ethanolic extract was completed to 1 mL with ethanol then mixed with 4 mL of distilled water or subsequently with 0.3 mL of 5% NaNO_2_ solution. After 6 min of incubation, 0.3 mL of 10% AlCl_3_ solution was added or then allowed to stand for 5 min, followed by adding 2 mL of 1 M NaOH solution to the mixture. The absorbance was detected at 415 nm using a spectrophotometer (UNICAM UV300—UK model). The quarcetin equivalent (QE) was used as a standard curve and the results were expressed as mg of QE per g dry weight (d.w) of the extract [33].

#### 2.9.7. Antioxidant Activity

##### DPPH Free Radical Scavenging Activity

The quantitative measurement of radical scavenging properties was carried out [34]. Briefly, 0.1 mM solution of 2,2-diphenyl-1-picryl-hydrazyl (DPPH) in methanol was prepared or 1 mL of this solution was added to 3 mL of each date palm ethanolic extract at various concentrations (50, 100, 150 and 200 µg/mL). Butylated hydroxyltoluene (BHT) was utilized as a positive control. Discoloration was measured at 517 nm after incubation for 30 min. The plot of scavenging activity on DPPH· was recorded and the IC50 value (concentration of the sample to scavenge 50% of the DPPH radicals; mg/mL) was then calculated.

The following equation was used to determine the radical scavenger capacity:

DPPH· scavenging effect (%) = [ADPPH − AS/ADPPH] × 100(5)

where ADPPH is the absorbance of the DPPH· solution or AS is the absorbance of the solution when the sample extract is added. 

##### Ferric Reducing Activity Power (FRAP)

Ferric reducing chelating activity of each treatment was assessed according to the previously described method [35]. Three milliliters from each extract at various concentrations, (0.5, 1 and 1.5 mg/mL) of ethanolic date palm extract were added to 60 µL of FeSO_4_ (2 mM). The reaction was started by adding 100 µL of ferrozine (5 mM). The mixture was mixed well or allowed to stand at room temperature for 10 min. Absorbance of the mixture was measured at 562 nm. Ethylenediaminetetraacetic acid (EDTA) was utilized as a positive control. The IC_50_ value (mg/mL), which is the concentration of the extracts that chelates 50% of the ferrous ion, was calculated from the nonlinear regression curve.

The inhibition % of the ferrozine–Fe^+2^ complex formations were calculated according to the following equation:

Ferric reducing activity (%) = [1 − A1/A0] × 100(6)

where A0 is the absorbance of the control (reaction mixture without samples) and A1 is the absorbance of the reaction mixture in the presence of the samples.

#### 2.9.8. Antioxidant Enzymes Assay (CAT, POX and PPO)

The fresh leaves (0.5 g) of date palm seedlings were extracted 30 days after NP foliar spray exposure by using 5 mL of phosphate buffer (50 mM, pH 7.7). The activity of catalase (CAT, EC: 1.11.1.6) was measured according to the previously reported method [36]. Peroxidase (POX, EC: 1.11.1.7) was detected by the method described earlier [37]. The activity was expressed as Unit. mg pro.^−1^ min^−1^. Polyphenol oxidase (PPO, EC: 1.10.3.1) activity was assayed according to the standard method [37]. For all the enzymatic calculations, the protein concentration was measured [38] utilizing bovine serum albumin (BSA) as the standard.

### 2.10. Statistical Analysis

All data are presented as means ± SD; all experimental groups and control groups were analyzed in parallel, in triplicates. Data were assessed by analysis of variance (ANOVA) through Duncan’s multiple range tests using SPSS software (SAS Institute Inc., Cary, NC, USA).

## 3. Results and Discussion

### 3.1. Characterization of Prepared Seaweed (Turbinaria triquetra) Nanopowders (NP) 

#### 3.1.1. Particle Size and Zeta Potential

**Figure 3:**
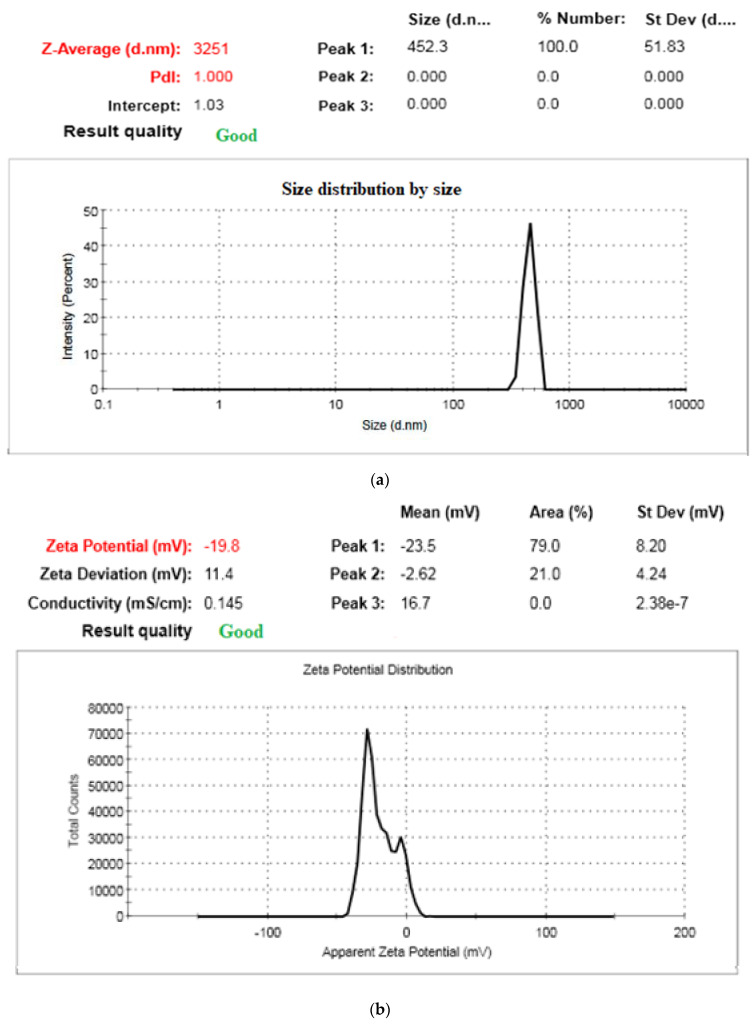
(**a**) Malvern Zeta sizer 3000 HSA size measurement of *T. triquetra* nanopowders. (**b**) Zeta potential of *T. triquetra* nanopowders.

Particle size or size distribution were detected utilizing the dynamic light scattering (DLS) method, which is the most common technique for detecting the size distribution profile of nanopowders (Figure 3a,b). The physiochemical parameters of the *T. triquetra* nanopowder showed a narrow size distribution and a small diameter (452 ± 20 nm). Zeta potential is a significant measure that represents the stability of the micellar system. It has been reported that under a relatively high surface charge, the particles can repel each other with a strong electrostatic repulsion force, thus enhancing the stability of the system [39]. Zeta potential is a measure of the magnitude of charges on nanopowders; values more positive than +30 mV and more negative than −30 mV demonstrate a strong stability to coalescence. In this test, the *T. triquetra* nanopowder particle displayed a zeta potential of about −23.5 ± 8.2 mV; hence, the *T. triquetra* nanopowder particle was stable. Additionally, in another work the authors described NPs with charges in the range of −25 and +25 mV as a sign of stability [40]. The above-mentioned contradictory findings on the relationship between zeta potential and particle size were likely caused by the fact that zeta potential depends on the process and material properties. Zeta potential’s absolute value and positive/negative charge are influenced by the material’s properties, including particle size, concentration, surface characteristics and ionic concentration, as well as by environmental variables such as pH and temperature. In this regard, Rajeshkumar et al., [41] used *Turbinaria conoides* as a reducing and stabilizing agent during the synthesis of gold nanoparticles since it possesses various natural ingredients, including fucoidan and polyphenolic compounds.

Additionally, the process of ball milling involves the movement of the balls, which imparts kinetic energy to the material being ground, breaks chemical bonds and produces fresh surfaces by fracturing material particles. In order to improve the properties that the raw material lacks, such as solubility, dispersion, surface effects and chemical reactivity, superfine grinding technology has developed [42]. The advantages of high energy ball milling are its flexibility, simplicity, ease of handling, capacity for large production runs, suitability for different materials and low cost [43].

#### 3.1.2. Fourier Transform Infrared (FT-IR) Spectra of the Seaweed Nanopowder of *Turbinaria triquetra*

**Figure 4:**
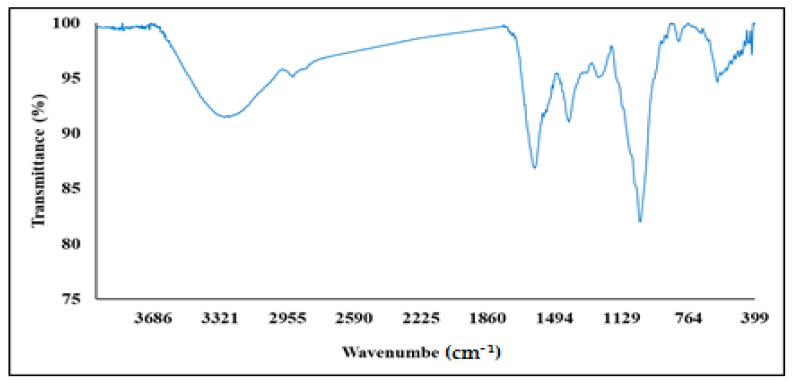
FT-IR spectra (400–4000 cm^−1^) of the transmittance of the nanopowder of *Turbinaria triquetra* (%).

The FT-IR spectral analysis was carried out to identify the potential biomolecules in the crude extract of the seaweed nanopowder. The FT-IR spectra (400–4000 cm^−1^) of the nanopowder of *Turbinaria triquetra* showed distinct peaks at different wave numbers (cm^−1^) which are related to the different functional groups of various chemical ingredients as presented in Table 1 and Figure 4. Generally, the FT-IR results suggested the presence of alkane, alkene, carboxylic acids, alcohols, amines, nitro compounds, phenols and ketones. These results are in harmony with those of various medicinal plants [44,45]. Moreover, the FT-IR results indicated the presence of different functional groups in the nanopowder of *Turbinaria triquetra*, especially the negatively charged groups such as O-H, N-O and C=O; these groups explain the negative charge of zeta potential (about −23.5 ± 8.2 mV) of these nanoparticles. The biomolecules found in the marine brown seaweed *Turbinaria triquetra* had a variety of chemical bonds and functional groups according to FT-IR spectrum research. The FTIR results showed that the compounds present in the marine brown seaweed *Turbinaria triquetra* were aromatic, alkanes, amides or amines and may function as potential stabilizers of nanoparticles in aqueous media. These findings are well supported by the literature [46].

#### 3.1.3. UV Scan of the Nanopowder of *Turbinaria triquetra*

**Figure 5:**
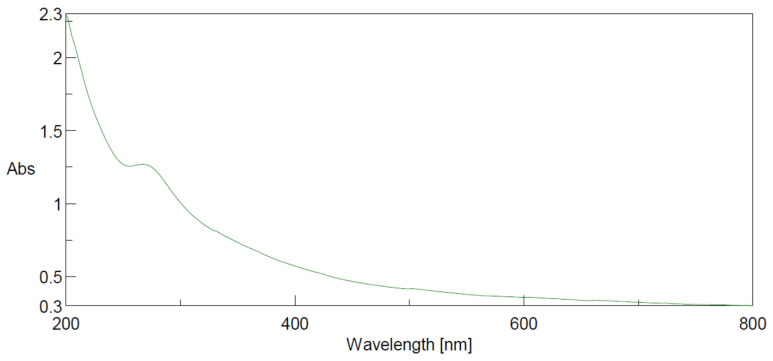
UV scan of the water-diluted suspension of the nanopowder of *Turbinaria triquetra*.

The UV scan of the water-diluted suspension of the nanopowder of *Turbinaria triquetra* showed a broad peak at about 270 nm (Figure 5). 

The peak obtained at 270 nm for the prepared sample is in accordance with earlier reports by Tungare et al. [47]. These peaks corresponded to the n–π* and π–π* transition of the –C=O and C=C bonds of conjugated π-domains.

This absorption peak may be associated with the characteristic absorption of chlorophyll pigments and flavonoids. The spectra for flavonoids typically lie in the range of 190–800 nm [48]

Using UV-VIS and FT-IR, Rajeswari and Jeyaprakash [49] assessed the bioactive compounds found in the brown seaweed *Sargassum wightii*. The findings demonstrated that the flavonoids’ characteristic spectral bands consisted of two absorption spectra with peak values in the ranges of 230–290 nm and 300–360 nm. These maxima’s actual position and perceived intensities provide extremely useful information about the nature of flavonoids. The existence of phenolic and alkaloids chemicals in *Sargassum wightii* was then revealed by the peak that occurred at 234–676 nm.

### 3.2. TEM and SEM Characterizations 

#### 3.2.1. TEM Characterization 

**Figure 6:**
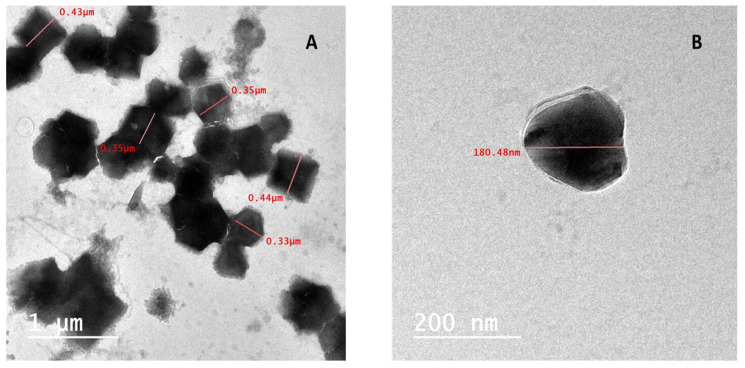
(**A**,**B**) TEM image of prepared seaweed nanopowders.

TEM were utilized to observe the particle size and shape of nanopowders. TEM detection using low and high resolution power displayed the output of hexagonal nano-seaweed with particle sizes between 180 and 450 nm, as shown in Figure 6A,B. 

The nanopowder had an average particle size of approximately 180 and 450 nm with a varied shape similar to hexagonal and pseudo-spherical shapes, according to the TEM micrograph. Moreover, this result of TEM reflected the results of the physiochemical parameters of *T. triquetra* nanopowder which showed a narrow size distribution and a small diameter (452 ± 20 nm) as presented in Figure 3a.

#### 3.2.2. SEM Characterization 

**Figure 7:**
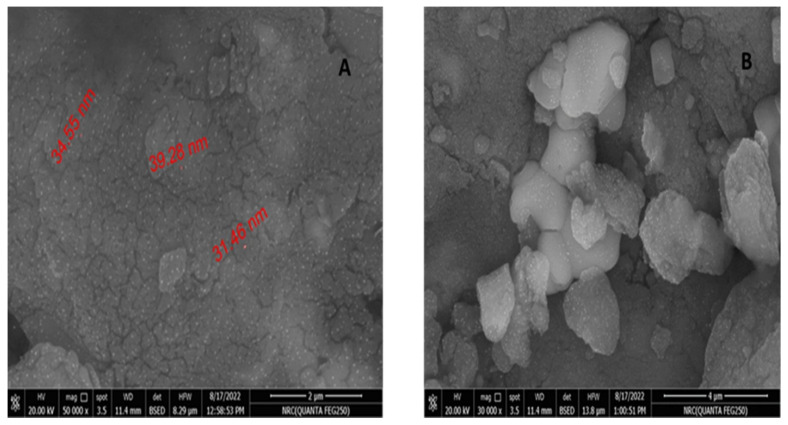
(**A**,**B**) SEM analysis of the image of prepared seaweed nanopowders.

The SEM micrographs of the prepared material are presented in Figure 7A,B. The prepared material exhibited an irregular particle shape without the formation of any bulky crystals. Moreover, the particles were agglomerated with non-uniform dimension. More importantly, nanopowders of seaweed can clearly be seen as shining dots on the external surface of the prepared material. The length of these nanopowders was measured to be in the range of 30–40 nm. 

It was shown that most of the nanopowders were similar to hexagons with some agglomerated in shape and the particle diameter ranged from 31.46 to 39.28 nm with some deviations. SEM analysis outlined the morphology and size of the nanoparticles that were dispersed moderately in the medium. The obtained results are similar to those in earlier published work [50].

At the nanoscale to micrometer scale, morphological characterization is frequently carried out using electron microscopy techniques such as SEM or TEM. Resolution of the TEM is 1000 times greater than that of the SEM [51]. Interactions between bio-compounds, such as polysaccharides, proteins, polyphenols, phenolic compounds or metal atoms can affect the size and structure of the resulting nanoparticles [52]. The size of nanoparticles is typically accepted to be between a few nanometers and <1000 nm [53]. A solid or dispersed particle with a size between 10 and 1000 nm has also been included in the definition of a nanoparticle by different researchers [54,55]. Nanoscale particles are included in the particle size range of 100–1000 nm, but not microscale particles because the term “microparticle” refers to particles with a dimension of 1–1000 μm [56]. Many authors have used the term “nanoparticle” in relation to their materials of sizes >100 to 1000 nm, such as 150–250 nm [57], 234.7 nm–892.6 nm [58], 333 nm [59] and 18 ± 4 nm [60]. Agriculture is one area where nanotechnology is being used more and more specially in the field of biofertilizers. It might be challenging to predict the outcome of a particular NP’s interaction with a live system [61]. The NP’s physicochemical properties are the primary factor in the effects that are produced [62]. The qualities of morphology, surface charge, concentration and size distribution are examples of those that, when changed separately, can have a variety of impacts in the same system. Probably, the TEM image revealed the internal structure and realistic particle sizes because of the obvious generally high prospects and the projected electrons’ energization to emerge into the sample. SEM, however, only scans the subjected sample region in this situation. Accordingly, this makes researching actual particle sizes challenging and results in some inconsistencies when compared to TEM examination. Additionally, the particle size was different in SEM and TEM results, we guess it is a matter of resolution under the set zoom in case of SEM. The image could demonstrate the aggregates of the primary particles. Another thing, if our material is not uniform as it is an organic sample (seaweed) that can exhibit different size distribution profiles calculated from images.

#### 3.2.3. XRD Characterization

**Figure 8:**
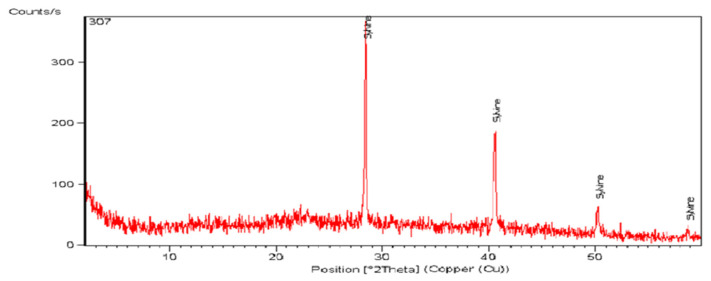
XRD pattern of the prepared seaweed *Turbinaria triquetra* nanopowder.

The X-ray diffraction patterns exhibited in Figure 8 explain the crystallinity and phase purity of *Turbinaria triquetra.* The *Turbinaria triquetra* contained sharp and narrow diffraction peaks, indicating a high crystallinity structure. The diffraction peaks of prepared seaweed *Turbinaria triquetra* nanopowders were found at 2 θ = 28.367°, 40.5761°, 50.2454° and 58.7005°. Moreover, the XRD pattern revealed the presence of KCl in the form of Sylvine with Semi Quant. (100%).

These results are in agreement with Żelazny and Jarosiński [63], who used the X-ray diffraction pattern to characterize fly ash fertilizer and they observed that potassium was found in the form of KCl (Sylvine) with the reference code (01-076-3368).

### 3.3. HPLC Profile of Prepared Seaweed Nanopowders

**Figure 9:**
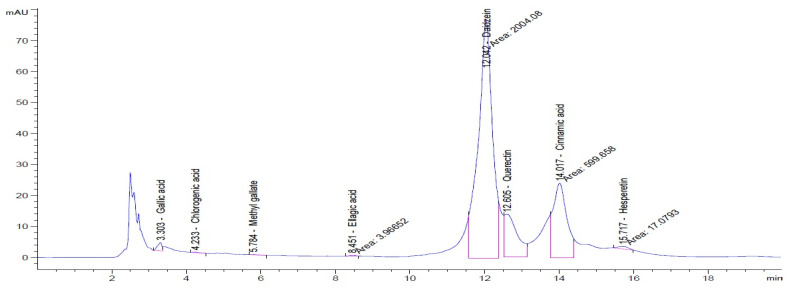
HPLC chromatogram of the methanolic extract of the seaweed nanopowders.

To clarify the polyphenolic profile, the major polyphenolic compounds in the methanolic extract were identified using HPLC analysis. By comparing the authentic standards, the components of cinnamic acid, gallic acid, ellagic acid, chlorogenic acid, methyl gallate, daidzein, quercetin and hesperetin were observed in the methanolic extract, as shown in Table 2 and in Figure 9.

One of the largest distributed categories of phytochemicals in seaweed is the polyphenolic compounds. They are considered as protective agents that are created in response to various stimuli and are components of defensive mechanisms against herbivory [2,7]. Accordingly, Table 2 reveals the presence of a number of important compounds such as daidzein and quercetin which had the highest levels (1212.027 and 402.647 µg/g, dw), respectively, whereas chlorogenic acid and methyl gallate represented the lowest levels (2.159 and 0.657 µg/g, dw), respectively. It was revealed that a number of seaweed species contained flavonoids such as quercetin, rutin and hesperidin. For instance, *Sargassum muticum* or *Sargassum vulgare* are capable of producing isoflavones such as daidzein or genistein [64]. Based on the current findings, it is clear that nanopowders of seaweed (*Turbinaria triquetra*) could be used in industry applications as an effective natural source of antioxidants or as an antibacterial agent due to their high content of valuable polyphenolics. The findings of the present study will be valuable for further investigations on *Turbinaria triquetra* aiming to isolate, describe and identify the particular phenolic compounds for their industrial applications. For example, daidzein (4′, 7-dihydroxyisoflavone) has great biological effects due to its anti-inflammatory and anticancer properties. Additionally, phenolic components such as phenolic acids, flavonoids, isoflavones, cinnamic acid, quercetin and ellagic acid were present in the extracts. Because of their well-documented beneficial effects on humans, they can be valuable constituents in medicines and feed additives, particularly by protecting plants from biotic or abiotic stress (antibacterial activity, scavenging of free radicals, host defense activity, etc.) as reported previously [65]. Some commercial seaweed fertilizer contains phenolic compounds, which have anti-microbial activity and may act as a barrier against plant diseases [66].

### 3.4. Elemental Composition of Seaweed Nanopowders

The mineral composition of NP powders was detected, and the obtained results are recorded in Table 3. Natural nanoparticles provide a variety of biological functions, such as antimicrobial or antifungal properties, plant growth enhancers or have capacity to improve crop productivity and quality [67,68]. Materials used to manufacture nanofertilizers often belong in two categories: metal oxides such as titan oxide, manganese oxide, iron oxide, carbon nanotubes and synthetic polymers and natural polymers such as chitin, chitosan, seaweed, etc. Metal oxides and synthetic polymers have the problem of not being biocompatible or biodegradable [69]. Generally, numerous topographical factors, such as temperature, water salinity, amount of light intensity and nutrient availability have a significant impact on the mineral composition of seaweeds. Due to these environmental factors, various physiological and metabolic processes in seaweeds are either stimulated or inhibited. Marine algae have more than 65 trace elements in a high concentration compared to terrestrial plants [70]. Additionally, the value of edible seaweeds in human nutrition is based on their content of several minerals, including sodium (Na^+^), magnesium (Mg^+2^), potassium (K^+^), calcium (Ca^+2^) or chloride (Cl^−^) [71]. Seaweeds are a rich source of micronutrients; their content in biomass is more than 40%. This is due to the fact that seaweeds concentrate metal ions as carbonate salts in their fronds after collecting them from saline water [72]. In Table 3, the data revealed that NP contained higher concentrations of selenium (41 µg/g), manganese (54.0 µg/g) and zinc (165.6 µg/g) which is consistent with previous observations for the same seaweed genera [72]. Nickel may be necessary for the maintenance of membrane structure, the metabolism of nucleic acids or as an enzyme cofactor. The concentration of Ni in the seaweed sample reached 41.6 µg/g. In contrast, hazardous metals such as arsenic (As), copper (Cu) and cadmium (Cd), may accumulate in seaweeds at levels up to 100–200 times higher than in land plants. This fact is crucial to keep in mind when thinking about using seaweed as a fertilizer because it may pose a risk to human health. Furthermore, it is important to remember that the hazardous metals’ harmful effects depend on their physical nature. For instance, (As) is more poisonous in of the inorganic form and less toxic when present in seaweeds in the organic form.

In agro-ecosystems, seaweeds can successfully replace chemical fertilizers and pesticides by dramatically enhancing soil fertility, which improves plant growth or protection by increasing nutrient availability. They can also generate bioactive compounds such as phytohormones, which help to develop root networking and protect plants from pathogens and pests [2]. The most widely utilized technique in the agriculture sector is liquid algae biofertilizers. These liquid fertilizers primarily contain high trace element concentrations and chemicals that regulate plant growth hormones (particularly cytokinins). Algal extracts contain several critical elements, such as Zn, K, Mg, Ca, P, S, K, Zn, Mo, Cu, Co or Fe, as well as a number of growth regulators, vitamins or polyamines that increase nutrient status, vegetative growth, yield or fruit quality in plants [73]. Moreover, potassium (K) content in seaweeds plays a significant role in the opening and closing of the stomata, in cell division stimulation and in the transfer of saccharides and starches between different organs of plants [74].

### 3.5. Vegetative Growth of Treated Date Palm Seedlings

#### Shoot Height and Leaf Area

**Figure 10:**
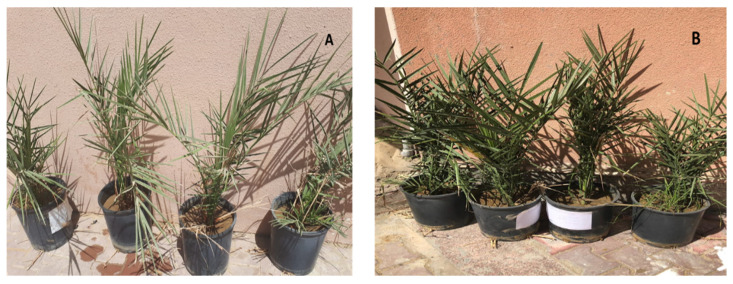
Date palm seedling (**A**) at zero time treatments and (**B**) after two weeks of treatments.

Shoot height (cm), leaf area (cm^2^), number of branches per plant and dry weights of leaves (g) were increased significantly by increasing the applied doses of 25, 50 and 100 mg L^−1^ seaweed liquid NP. Under 50 mg L^−1^ (T3) or 100 mg L^−1^ (T4), the values of plant height (45.8 ± 0.09 and43.3 ± 0.04, respectively) were the highest compared to the value of T1 and T2 (34.2 ± 0.11 and 38.4 ± 0.32) (Figure 10 and Table 4).

Similar trends were observed for the number of branches per plant. The maximum numbers of branches (10.0 ± 0.4 and 9.0 ± 0.23) were counted in both (T3 and T4, respectively). Moreover, the application of nanopowders significantly increased the dry weight at T3 (12.0% ± 0.22) compared to the control plant T1 (4.0% ± 0.21). Due to the ability of the nano-molecules to penetrate the cytoskeleton and improve the efficiency of the cell membrane, the usage of biomass and dry weight parameters during the vegetation period aided the understanding of the cell division and elongation. The leaf area is also an important growth parameter used to evaluate the quality of date palm seedlings. Leaf area of the control treatment (T1) was found to be 170.5 ± 0.2 cm^2^/plant; this increased by 320% at T3 as the value was 545.9 cm^2^/plant compared with the control group.

From Table 4, it is shown that the foliar application of prepared seaweed nanopowders improved leaf area, number of branches and the percentages of dry weights. In this concern, seaweed extracts are inexpensive, good for the environment because they do not pollute water, soil, or air and they are abundant in nutrients, growth regulators, vitamins and organic acids. They are also easy for plants to absorb [75]. An increase in the number of leaves and the net weight of the plants would follow an increase in plant height. Photosynthesis takes place in the leaves of the plant. As the ability for photosynthesis expands, an increase in the number of leaves would enhance photosynthesis as well [76].

### 3.6. Preliminary Phytochemical Screening

Generally, the identification of phytochemical bioactive compounds in plants is crucial for the purification process, separation and detection of metabolites, and it may be critically thought of as a replacement strategy to understand plant infections. 

Table 5 represents the qualitative analysis of some phytochemical constituents that were identified in date palm seedlings treated with four different foliar treatments of NP. Different phytochemical compounds in the extract such as, alkaloids, terpenoids, steroids, phenolic, tannins, saponins and flavonoids were tested. It was observed that most of them gave highly positive values at T3 and T4 in both solvents except for the saponins. In accordance with our results, different extracts of one seaweed species named *Turbinaria ornata* have been evaluated and different bioactive compounds such as alkaloids, tannins, terpenes, phenols, saponins, flavonoids, quinones, carbohydrates, coumarins, alkaloids, steroids, terpenoids and cardiac glycosides were identified [77]. The author suggested that these bioactive compounds in seaweed extract have antioxidant and anti-proliferative effects.

Both ethanolic and di-ethyl ether extracts contained phenolic, but the ethanolic extract was still higher than the semi-polar solvent di-ethyl ether. It was revealed that the di-ethyl ether extract had a low saponins component (+). On the other hand, the effect of both organic and non-organic fertilizers on the mitigation of secondary metabolites in *Coriandrum sativum* was evaluated. The preliminary phytochemistry found coumarins, flavonoids and steroids in the extract and there were no differences in the type of phytochemistry when the plant was treated with organic or non-organic fertilizers [78]. Additionally, seaweed extracts (SE) known as biostimulants that are produced from seaweed, particularly brown and red algae, have been shown to improve plant stress resistance and promote plant growth. SE mainly contain natural hormones, such as auxin, cytokinins, gibberellin, abscisic acid or other bioactive substances such as polysaccharide, betaine and phenolic compounds which are considered to be bio-stimulating in crop production [79]. The unique physical, chemical and biological properties of phenolic compounds make them suitable for use as antioxidant agents. Antioxidant properties of brown seaweeds are greater than that of red or green ones [80]. The di-ethyl ether extract contained a higher (+++) of steroids than the ethanol extract (++) at T3. Terpenoids have polar groups, whereas the di-ethyl ether solvent is a semi-polar solvent (+) compared to the ethanolic extract (+) at T3 one; hence, the terpenoids molecule was identified at low levels in the di-ethyl ether solvent. It is well known that the foliar spray of organic seaweed nanopowder extracts increases the vegetative growth of plants together with increases in plant height, total chlorophyll content and leaf area index because brown seaweed extract is a very good source of phytochemicals that stimulate plant growth [67]. This is most likely because nanoparticles have the benefit of being able to stimulate specific metabolic processes that are typically activated during the early germination growth [67].

### 3.7. Chlorophyll Content

Based on the results presented in Table 6, chlorophyll *a*, chlorophyll *b*, total chlorophyll *a+b* or total carotenoids significantly affected the foliar application of the seaweed nanopowders on date palm seedlings specially for the T3 and T4 treatments. Application of seaweed on the date palm seedlings had a significant effect on chlorophyll *a+b* content especially at T3 and T4 which gave the highest values of 9.64 ± 0.07 and 8.50 ± 0.12 mg. g^−1^ FW, respectively. On the other hand, T1 (no seaweed spraying) gave the lowest value (4.4 ± 0.07 mg·g^−1^ FW). The application of seaweed led to a higher chlorophyll concentration than non-treated treatments, which could be explained by the fact that seaweed extracts contain betaine which has the ability to slow down chlorophyll breakdown. By activating specific photosynthesis-related enzymes, the metal elements have a direct or indirect impact on the green cells’ capacity to fix carbon dioxide; this indicates the development of new cells and tissues. Nevertheless, the foliar application of one seaweed named *Caulerpa scalpelliformis* in the form of liquid fertilizer enhanced the contents of the pigments of the black gram (*Vigna mungo* L.) plant because seaweed fertilizer contains different bioactive compounds [81]. In our work, seaweed nanopowders contained both macro elements (Ca and Mg) and microelements (Fe, Zn, Cu, and Mn) as presented previously in Table 3. In accordance with our results, these elements are required for cell division and elongation as well as for respiration, photosynthesis and other metabolic functions [82].

The obtained results are in agreement with the previous studies that stated that biofertilizers and iron nano-oxides are capable of promoting different physiological parameters in maize plants when grown under drought stress [83]. The nanoparticles simply diffuse through the stomata and penetrate the vascular bundles when delivered as a foliar spray. They move inside the plant by following apoplastic pathways. The building blocks of date palm tissues are thought to be the monosaccharides that are directly created during photosynthesis [84]. Generally, the overall carotenoid concentration of dates varied depending on whether they were yellow or red in colour. Numerous processes, including germination, antioxidant activity, macro- or micronutrients, chlorophyll content, chloroplast quantity or photosynthesis in plants are affected by how the nanoparticles are delivered to the plant [85].

### 3.8. Polyphenolic Contents

The amount of bioactive compounds that known to improve health, such as polyphenolic compounds in date palm seedlings, were evaluated (Table 7). Seedling plants sprayed with T3 and or T4 exhibited significant increases in both phenolic and flavonoid content when compared to T1 or T2. Total phenolic contents varied from 6.31 ± 0.31 to 14.02 ± 0.2 mg GAE/g (d.w). Moreover, flavonoid content ranged from 4.31 ± 0.22 to 12.02 ± 0.05 mg QE/g (d.w).

So, it is clear that the foliar application of nano-seaweed may enhance the concentration of total phenolic and flavonoid even when there is no chemical fertilizer. Moreover, total soluble sugar contents were evaluated when grown under different doses of the nanoparticles of the seaweed extracts. It was shown that there was an increase in total soluble sugars from 30.8% ± 1.4 at T1 to 41.9% ±1.5 at T2. 

In the Arab East, date palm fruits are valued for both their great nutritional content and their therapeutic applications. The notable antioxidant activity of the date palm extracts could be responsible for the varied amounts of phenolic and flavonoid components. The increase in the bioactive compounds in date palm seedlings in response to the foliar application of seaweed nanopowders may be explained as follows: the superfine powder prepared using the ball-milling technique released greater contents of the cell-wall-bound bioactive compounds because of the large surface area and the triggering of partial cellular degradation that resulted in more soluble forms of the resultant metabolites. The use of ball milling technology may accelerate the breakdown of cell walls, increasing the release of chemicals bound to cell walls and those that work as biofertilizers. The application of seaweed nanofertilizers on date palm trees increased both total sugars and reduced the percentage of sugars, which makes them a wonderful source of energy [86].

### 3.9. Antioxidant Activities

There are numerous processes usually used to detect antioxidant activity. In our experiment, two different approaches were employed: the radical scavenging capability of 2,2 diphenyl-1-pikryl-hydrazyl (DPPH) method or the FRAP (ferric-reducing antioxidant power) which relies on the capacity of the sample to chelate metals. 

#### 3.9.1. The DPPH Activity (%) 

**Figure 11:**
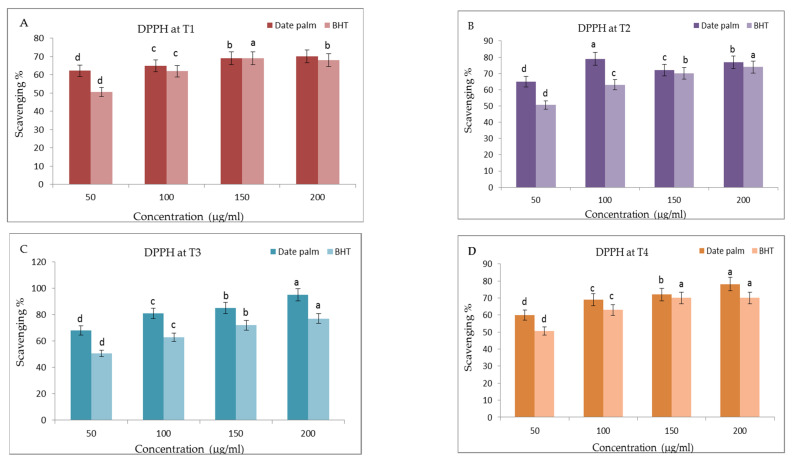
(**A**–**D**) Effect of prepared of seaweed nanopowders (T1, T2, T3 and T4) on 2,2-diphenyl-1-picryl-hydrazyl (DPPH·) scavenging activities of date palm (*Phoenix dactylifera* L.) seedlings compared to Butylated hydroxyltoluene (BHT). Vertical bars on columns represent mean ± SD (*n* = 3). Different letters indicate significant differences among the treatments according to Duncan’s test (*p* ≤ 0.05).

The response of date palm seedlings to different applied doses of liquid nano-seaweed (T1, T2, T3, T4) during the growth period was represented by an increase in DPPH antioxidant activity (Figure 11A–D). The DPPH % values were significantly increased in the T3 treatment, which reached 95.1% compared to the control one T1 (70.2%) at the same concentration (200 µg/mL) of the date palm extract. Moreover, T4 treatment also caused a significant increase in DPPH value (78.1%) when compared with the control one T1 (70.2%) at the same concentration (200 µg/mL) of the date palm extract. The mean inhibitory concentration (IC_50_) of the date palm aqueous extract and BHT at T3 was found to be 64.19 µg/mL and 36.44µg/mL, respectively. At T4, date palm seedlings possessed an antioxidant activity in relation to DPPH radicals with an IC_50_ of 47.56 µg/mL when the IC_50_ of BHT was 35.46 µg/mL. With respect to state of the art works, it has been shown that the molecules potentially have antioxidant activities for an IC50 < 500 µg/mL [87]. With respect to the present sample showing an IC_50_ < 100 µg/mL, in previous research performed by Bentrad and Gaceb-Terrak, [88], since the lowest IC_50_ reveals the most significant antioxidant activity when compared to other standard antioxidants, the antioxidant activity is actually directly related to the extract concentrations [88].

In order to remove reactive oxygen radicals (ROS) from food systems and consequently, from the human body, it is critical to have a strong H_2_O_2_ scavenging activity. However, this activity (95.1%) which was detected in the T3 treatment was still higher than that of the synthetic antioxidant BHT (77.1%) at the same concentration (200 µg/mL). In this concern, the preparation of superfine leaf powders of *Quercus salicina* (Blume) by using the ball milling technique enhanced the antioxidant activity and this technique is well used in the manufacturing of active food ingredients [89].

Moreover, in this concern, the green biosynthesized silver nanoparticles from the *Brachychiton populneus* leaf extract were found to have a high antioxidant activity when assayed by the DPPH method [90]. It could be concluded that plants are affected physiologically and morphologically by the foliar application of nanopowders that enter the cell from above-ground organs (cuticle, epidermis and stoma). When a particle is 40 nm or less, it can be transported by the stomata and carried by the phloem to other areas of the plant.

#### 3.9.2. Ferric-Reducing Antioxidant Activity Power (FRAP)

**Figure 12:**
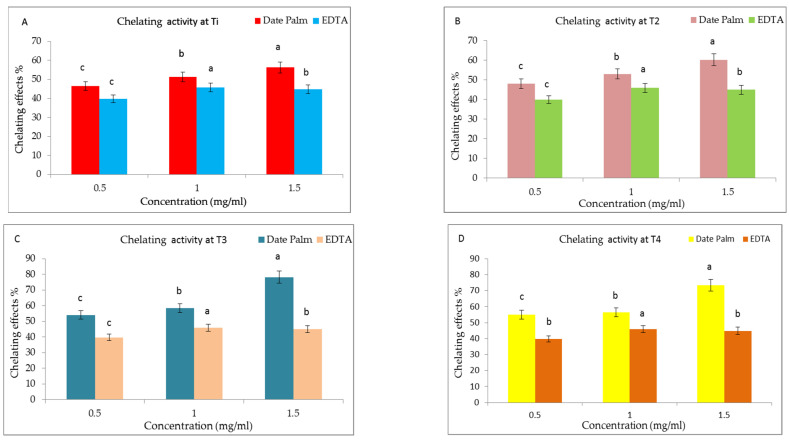
(**A**–**D**) Effect of prepared of seaweed nanopowders (T1, T2, T3 and T4) on the ferric-reducing antioxidant power of date palm (*Phoenix dactylifera* L.) seedlings compared to EDTA. Vertical bars on columns represent mean ± SD (*n* = 3). Different letters indicate significant differences at the 5% level (Duncan’s multiple range tests at *p* ≤ 0.05).

The chelating of metal ions is the main method used to minimize ROS formation, which is related to redox active metal catalysis. Figure 12A–D displays the results of the chelating scavenging activity in date palm seedlings after 30 days grown under different treatments (T1, T2, T3 and T4), respectively.

In the T3 treatment, the ferric-reducing antioxidant activity of the date palm extract reached to the maximum value (78.3% ± 0.14) at 1.5 mg/mL which was higher than the positive standard EDTA (44.9% ± 0.21) at a concentration of 1.5 mg/mL, whereas the FRAP activity percent (%) in the T4 treatment was 69.3.4% ± 0.12 at a concentration of 1.5 mg/mL which was still higher than the positive standard EDTA (44.9% ± 0.21 at the same concentration (1.5 mg/mL). The mean inhibitory concentrations (IC_50_) of the date palm aqueous extract and standard EDTA at T3 were found to be 0.3 mg/mL and 1.46 mg/mL respectively. The impact of different kinds of seaweed liquid fertilizer seaweeds such as *Ulva eticulata, Ulva lactuca, Gracillaria corticata, Kappaphycus alvarezii, Padina pavonica and Sargassum johnstonii* on DPPH antioxidant activity was previously investigated [91]. The author stated that the seaweed liquid fertilizer improved the antioxidant activity and also antioxidant enzymes such as catalase and peroxidase of the vegetable plant. In accordance with our findings, the applications of organic fertilizer improved the antioxidant activity as measured by the DPPH and FRAP assays whereas the inorganic fertilizer was shown to decrease the antioxidant activity in the *Kacip Fatimah* (*Labisia pumila* Benth) plant [92]. In fact, plants frequently produce antioxidant bioactive compounds in order to guard against or avoid abiotic and oxidative damage.

### 3.10. Enzyme Activity (CAT, POX and PPO)

**Figure 13:**
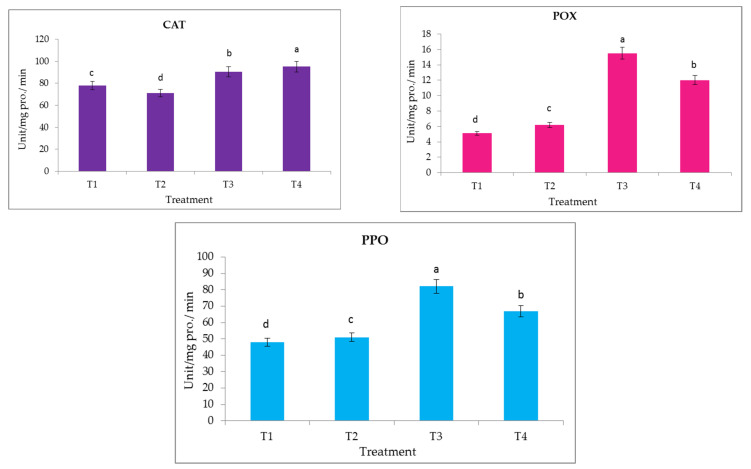
Effect of prepared of seaweed nanopowders (Abbreviations: T1, T2, T3, and T4 represent control, 25, 50, and 100 mg L^−1^ nano-seaweed, respectively) on CAT, POX and PPO (U. mg pro^−1^ min^−1^) of date palm seedlings. Vertical bars on columns represent mean ± SD (n = 3). Different letters indicate significant differences at the 5% level (Duncan’s multiple range tests at *p* ≤ 0.05).

The response of the date palm seedlings to different applied doses of liquid nano-seaweed exhibited a significant increase in enzymatic antioxidants including CAT, POX and PPO, as shown in Figure 13. It is clear that previous enzymatic antioxidants (CAT, POX and PPO) were significantly increased by increasing the applied doses of nano-seaweed. The highest values of CAT, POX or PPO enzyme activities were achieved when both applied doses of T3 and T4 nano-seaweed were sprayed on the date palm seedlings.

Although reactive oxygen species (ROS) are important for the plant defense mechanism, their accumulation can cause oxidative harm. Following exposure to environmental stress, the activity of the antioxidant enzymes such as POD, APX and CAT may be increased. These enzymes play a crucial role in detecting and detoxifying excess reactive oxygen species. We observed a significant increase in the activity of CAT occurring after 30 days at T3 and T4 respectively. The values were 95.2 U mg pro^−1^ min^−1^ for T3 and 90.4 U mg pro^−1^ min^−1^ for T4 at day 30, respectively. However, values of the enzyme activity of T2 were similar to T1. A similar trend was observed for POX and PPO. Our findings are in agreement with other results [91,92,93] which reported that the simultaneous increase in the activity of these antioxidant enzymes contributes to an increase in the application of brown seaweed liquid fertilizer. Similar observations were observed in the onion plant when treated with the foliar application of exogenous antioxidant compounds [94,95,96,97,98,99,100,101,102,103]. The author found an increase in POX activity when the plant was treated by alpha tocopherol. Increases in these enzymes may have assisted the plant in destroying the H_2_O_2_ that was present under either normal or abnormal conditions and in maintaining the ascorbate pool, both of which increased the plant’s tolerance and maintained its optimal growth [104].

## 4. Conclusions

Our study showed that the prepared nanopowders of *Turbinaria triquetra* used in the form of a suspension solution stimulated different growth parameters of date palm seedlings (*Phoenix dactylifera* L. cv), Sukary. The characterization of the prepared nanopowder was completed with the help of UV–Vis spectrophotometer, FTIR, SEM, TEM and XRD. Particle size distribution analysis indicated that the average nanoparticle displayed a zeta potential of about −23.5 ± 8.2 mV; hence, the *T. triquetra* nano-powder particle was stable. Moreover, the FTIR analysis of the NP sample revealed the various functional groups involved in the seaweed sample. Moreover, nanoparticles contained different nutrients such as K, Cu, Fe, Mn, Se, Cr, Co, Ni, Br, Rb, Sr, Mo and Zn at different concentration. The HPLC profile of polyphenolic compounds revealed the presence of a number of important compounds such as Daidzein and Quercetin (1212.027 and 402.647 µg/g, dw, respectively). The foliar application of nanopowders in the form of a solution resulted in an enhanced leaf area, number of branches, dry weight, total soluble sugars and some other secondary metabolites. Additionally, the effects of nanopowders on the stimulation of chlorophylls, antioxidant activity (DPPH and chelating activity) and some antioxidant enzyme activities (POD, APX and CAT) were also observed. In most cases, the best effects were observed at 50 and 100 mg L^−1^ of the prepared seaweed solution. The isolation and purification of the compounds from seaweed (*Turbinaria triquetra*) may be useful for the future production of nanoparticles in large-scale trials. Moreover, the unique properties of seaweed nanopowders may be highly beneficial in the stimulation of plant growth, but, as their mechanisms of action are not fully understood, further detailed, biochemical and molecular studies on the impact of nanopowders on plant health, food safety and further experiments should be completed using different conditions.

## Figures and Tables

**Table 1:** The FT-IR fingerprint (Wavenumber, cm^−1^) of the nanopowder of *Turbinaria triquetra*.

No.	Wavenumber (cm^−1^)	Functional Group	Type of Vibration
1	604	C-Br (halo compound)	Stretching
2	815	=C-H (alkene)	Bending
3	880	C-H (alkane)	Bending
4	904	=C-H (alkene)	Bending
5	1026	C-N (aliphatic amines)	Stretching
6	1254	C-N (aromatic amines)	Stretching
7	1415	O-H (carboxylic acid)	Bending
8	1537	N-O (nitro compound)	Stretching
9	1603	N-H (amine)	Bending
10	1722	C=O (aliphatic ketone or carboxylic acid)	Stretching
11	2922	O-H (alcohol)	Stretching
12	3297	O-H (carboxylic acid)	Stretching
13	3684	O-H, free hydroxyl (alcohols, phenols)	Stretching

**Table 2:** Major bioactive compounds (µg/g, dw) identified by HPLC in methanolic seaweed nanopowders.

No	Bioactive Compounds	Area	Conc. (µg/g, dw)	Structure
1	Cinnamic acid	599.66	114.460 ± 0.63	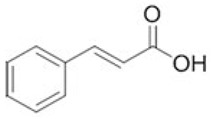
2	Gallic acid	22.18	16.619 ± 0.33	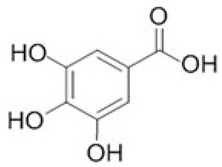
3	Ellagic acid	3.97	10.348 ± 0.21	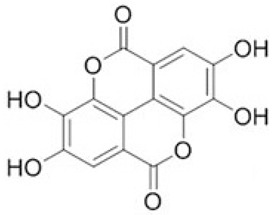
4	Chlorogenic acid	1.52	2.159 ± 0.14	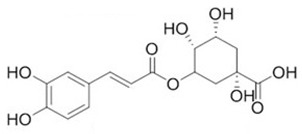
5	Methyl gallate	1.17	0.657 ± 0.03	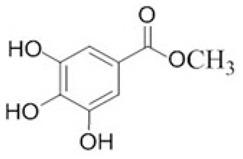
6	Daidzein	2004.08	1212.027 ± 1.75	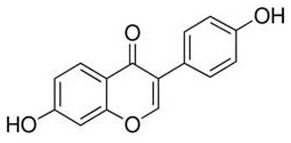
7	Quercetin	332.64	402.647 ± 0.81	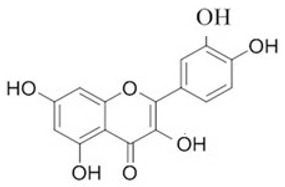
8	Hesperetin	17.08	9.291 ± 0.54	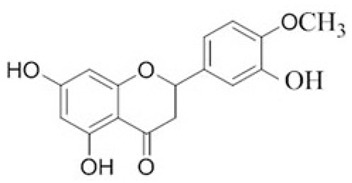

Values are means ± standard deviation (SD).

**Table 3:** Macronutrients and micronutrient contents of the *T. triquetra* seaweed nanopowder.

No	Trace Elements	µg/g	No	Trace Elements	µg/g
1	Se	41 ± 0.09	15	Ag	0.1 ± 1.00
2	V	0.9 ± 0.02	16	Cd	0.1 ± 0.01
3	Cr	8.9 ± 0.01	17	Sn	1.0 ± 0.02
4	Mn	54.0 ± 1.00	18	Sb	0.4 ± 0.04
5	Co	14.3 ± 0.07	19	Te	5.1 ± 0.04
6	Ni	41.6 ± 0.09	20	I	578.7 ± 0.05
7	Cu	4.3 ± 1.00	21	K	8.9 ± 0.08
8	Zn	165.6 ± 0.08	22	Ba	13.1 ± 1.00
9	Ga	2.2 ± 1.00	23	Si	378.94 ± 0.09
10	As	0.98.1 ± 0.02	24	Ti	299.75 ± 0.05
11	Br	684.2 ± 0.04	25	Al	211.2 ± 0.07
12	Rb	25.5 ± 1.00	26	Fe	279.6 ± 0.06
13	Sr	2140.6 ± 0.03	27	Mg	886.57 ± 1.00
14	Mo	0.6 ± 0.04	28	Ca	3162.08 ± 0.08

Values are means ± standard deviation (SD).

**Table 4:** Effect of the prepared seaweed nanopowders on some growth parameters of date palm (*Phoenix dactylifera* L.) seedling.

Growth Parameters	Seaweed Nanopowder Treatments			
	T1	T2	T3	T4
Plant height (cm)	34.2 ± 0.11 ^d^	38.4 ± 0.32 ^c^	45.8 ± 0.09 ^a^	43.3 ± 0.04 ^b^
Leaf area (cm^2^)	170.5 ± 0.2 ^d^	346.5 ± 0.12 ^c^	545.9 ± 0.11 ^a^	540.4 ± 0.05 ^b^
Number of Branches	5.0 ± 1.1 ^d^	7.0 ± 1.2 ^c^	10.0 ±0.4 ^a^	9.0 ±0.23 ^b^
Dry weight (%)	4.0 ± 0.21 ^d^	5.0 ± 0.16 ^c^	12.0 ± 0.22 ^a^	7.0 ± 0.13 ^b^

Each value in the table is mean ± standard deviation (*n* = 3). Where (T1: applying water as control, T2: 25 mg L^−1^, T3: 50 mg L^−1^, T4: 100 mg L^−1^ of seaweed liquid NP, respectively). Mean values in each column followed by different lower-case letters were significantly different according to Duncan’s multiple range tests (*p* ≤ 0.05). Different letters indicate significant differences among the treatments according to Duncan’s test (*p* ≤ 0.05).

**Table 5:** Preliminary phytochemical screening of the date palm seedlings after the foliar application treatments by prepared seaweed nanopowders.

Phytochemicals	Seaweed Nanopowder Treatments							
	Ethanol				Di-Ethyl Ether			
	T1	T2	T3	T4	T1	T2	T3	T4
Alkaloids	+	+	+++	++	+	+	++	++
Terpenoids	-	+	++	++	-	+	+	+
Steroids	+	-	+	+	+	+	+++	+
Phenolics	+	+	+++	++	+	+	+	+
Tannins	+	++	++	++	+	+	++	++
Saponins	-	-	+	+	-	+	-	-
Flavonoids	+	+	+++	++	+	+	+	+

(-) absent; (+) low; (++) moderate; (+++) high.

**Table 6:** Effect of the prepared seaweed nanopowder on the contents of pigments (mg/g FW) of date palm (*Phoenix dactylifera* L.) seedlings.

Pigments	Prepared Seaweed Nanopowder Treatments			
	T1	T2	T3	T4
Chlorophyll *a*	2.32 ± 0.1 ^d^	4.89 ± 0.11 ^c^	5.93 ± 0.20 ^a^	5.21 ± 0.09 ^b^
Chlorophyll *b*	1.08 ± 0.24 ^d^	1.87 ± 0.06 ^c^	3.71 ± 0.08 ^a^	3.29 ± 0.01 ^b^
Chl *a+b*	4.4 ± 0.07 ^d^	6.76 ± 0.04 ^c^	9.64 ± 0.07 ^a^	8.50 ± 0.12 ^b^
Carotenoids	0.8 ± 0.02 ^d^	0.9 ± 0.01 ^c^	1.6 ± 0.03 ^a^	1.3 ± 0.08 ^b^

Values are means ± standard deviation (SD) from three replicates. Mean values in each column followed by different letters are significantly different among treatments according to Duncan’s multiple range tests at *p* ≤ 0.05.

**Table 7:** Effect of prepared seaweed nanopowders on phenolic, flavonoid and total soluble sugar contents of date palm (*Phoenix dactylifera* L.) seedlings.

Bioactive Compounds	Prepared Seaweed Nanopowder Treatments			
	T1	T2	T3	T4
Total phenolic (mg GAE/g)	6.31 ± 0.31 ^d^	7.81 ± 0.11 ^c^	14.02 ± 0.2 ^a^	11.64 ± 0.8 ^b^
Total flavonoids (mg QE/g)	4.31 ± 0.22 ^d^	6.81 ± 0.17 ^c^	12.02 ± 0.5 ^a^	9.64 ± 0.1 ^b^
Total flavonoids/Total phenolic	0.68 ± 0.03 ^c^	0.87 ± 0.01 ^a^	0.85 ± 0.01 ^a^	0.83 ± 0.02 ^b^
Total soluble sugar (%)	30.8 ± 1.4 ^d^	41.9 ± 1.5 ^c^	66.1 ± 0.9 ^a^	56.9 ± 0.71 ^b^

Values are means ± standard deviation (SD) from three replicates. Mean values in each column followed by different letters are significantly different among treatments according to Duncan’s multiple range tests at *p* ≤ 0.05.

## Data Availability

Data are contained within the article.
